# Threshold effects of bone mineral density on mortality risk: a comprehensive analysis of BMI-mediated pathways in older population

**DOI:** 10.3389/fendo.2025.1567047

**Published:** 2025-07-22

**Authors:** Zifei Yin, Chen Kuang, Feng Gao, Feng Xu

**Affiliations:** Department of Orthopedics, Kunshan Traditional Chinese Medicine Hospital, Kunshan, China

**Keywords:** bone mineral density, body mass index, all-cause mortality, mediation analysis, older adults, NHANES

## Abstract

**Background:**

The precise relationship between bone mineral density (BMD) and all-cause mortality in older adults remains incompletely understood. This study aimed to investigate the association between BMD and all-cause mortality and to explore the mediating role of body mass index (BMI) in adults aged ≥60 years.

**Methods:**

A cohort study was conducted using data from the National Health and Nutrition Examination Survey (2007–2010, 2013–2014, and 2017–2018), including 6,289 participants aged ≥60 years. The application of Cox proportional hazards models enabled the evaluation of the association between BMD and all-cause mortality, while causal mediation analysis was performed to assess the mediating effect of BMI.

**Results:**

This study revealed that among the 6,289 participants, 1,422 (22.61%) deaths occurred during the follow-up period. The findings showed that there was a J-shaped association between BMD and all-cause mortality, with an increased mortality risk observed as BMD decreased. Higher BMD was associated with lower mortality risk, with evidence suggesting both direct and BMI-related pathways. The total effect was strongest for total femur BMD (-0.056, P<0.0001), followed by intertrochanter (-0.061, P<0.0001), trochanter (-0.043, P<0.0001), and femoral neck (-0.025, P=0.002). BMI appeared to partially mediate the protective associations, with varying proportions observed across sites: femoral neck (24.18%), trochanter (12.83%), total femur (11.17%), and intertrochanter (9.20%). The pathway analysis revealed that BMI was found to partially mediate the association between BMD and all-cause mortality. These associations remained robust after adjusting for demographic, socioeconomic, and clinical confounding factors

**Conclusions:**

This study identified site-specific threshold effects of BMD on mortality and quantified the mediating role of BMI. The findings suggest that maintaining an optimal BMI may be associated with reduced mortality risk for individuals with low BMD. Integrated interventions targeting both bone density and body mass management could be more effective in reducing mortality risk among older adults with low BMD.

## Background

The relationship between bone mineral density (BMD) and all-cause mortality in older adults has garnered significant attention in recent years; however, this area of research remains complex and not fully understood. Numerous studies have indicated that lower BMD is associated with an increased risk of mortality, primarily due to its association with fractures and other health complications, such as osteoporosis (1). For instance, Lloyd et al. reported that a substantial number of older adults in the U.S. suffer from low femoral neck BMD, which is a critical indicator of osteoporosis and related fractures, thereby increasing mortality risk (2). Furthermore, the mechanisms underlying this association are multifaceted and may involve various factors, including body mass index (BMI), which is a widely recognized measure of body weight relative to height.

BMI has been observed to have a non-linear relationship with mortality, often represented as a J-shaped curve, particularly in older populations. Research has shown that a higher BMI within a certain range can be linked to lower mortality rates, a phenomenon known as the “obesity paradox” (3). This paradox suggests that older adults with higher BMI may experience increased survival rates despite the general health risks associated with obesity. For example, studies have demonstrated that older adults with higher baseline BMI tend to have lower mortality rates, even if their BMI decreases over time. This relationship may be influenced by factors such as muscle mass, fat distribution, and overall health status, complicating the interpretation of BMI as a sole indicator of health in older adults (4).

The interplay between BMD and BMI is particularly intricate. While obesity can lead to increased bone density due to mechanical loading and hormonal influences, it may also contribute to metabolic complications that adversely affect bone health. Weight loss in obese older adults has been shown to increase bone turnover and decrease BMD, which could exacerbate age-related osteopenia (5). Therefore, it is essential to investigate the direct and indirect pathways through which BMD and BMI influence mortality in older adults. This study aims to explore the association between BMD and all-cause mortality in adults aged ≥60 years, with a particular focus on the mediating role of BMI. By accounting for potential confounding factors such as demographic characteristics, socioeconomic status, and clinical conditions, we seek to provide a more nuanced understanding of the relationship between BMD, BMI, and mortality in this population.

## Methods

### Study design and population

This longitudinal cohort study utilized publicly available data from the National Health and Nutrition Examination Survey (NHANES), a nationally representative program conducted by the Centers for Disease Control and Prevention (CDC). NHANES employs a multistage, stratified probability sampling design to collect health-related data from the noninstitutionalized U.S. civilian population. Detailed descriptions of the NHANES survey design, data collection protocols, and analytical standards are available on the NHANES website. Ethical approval for NHANES was obtained from the National Center for Health Statistics (NCHS) Research Ethics Review Board, and all participants provided written informed consent.

**Figure 1:**
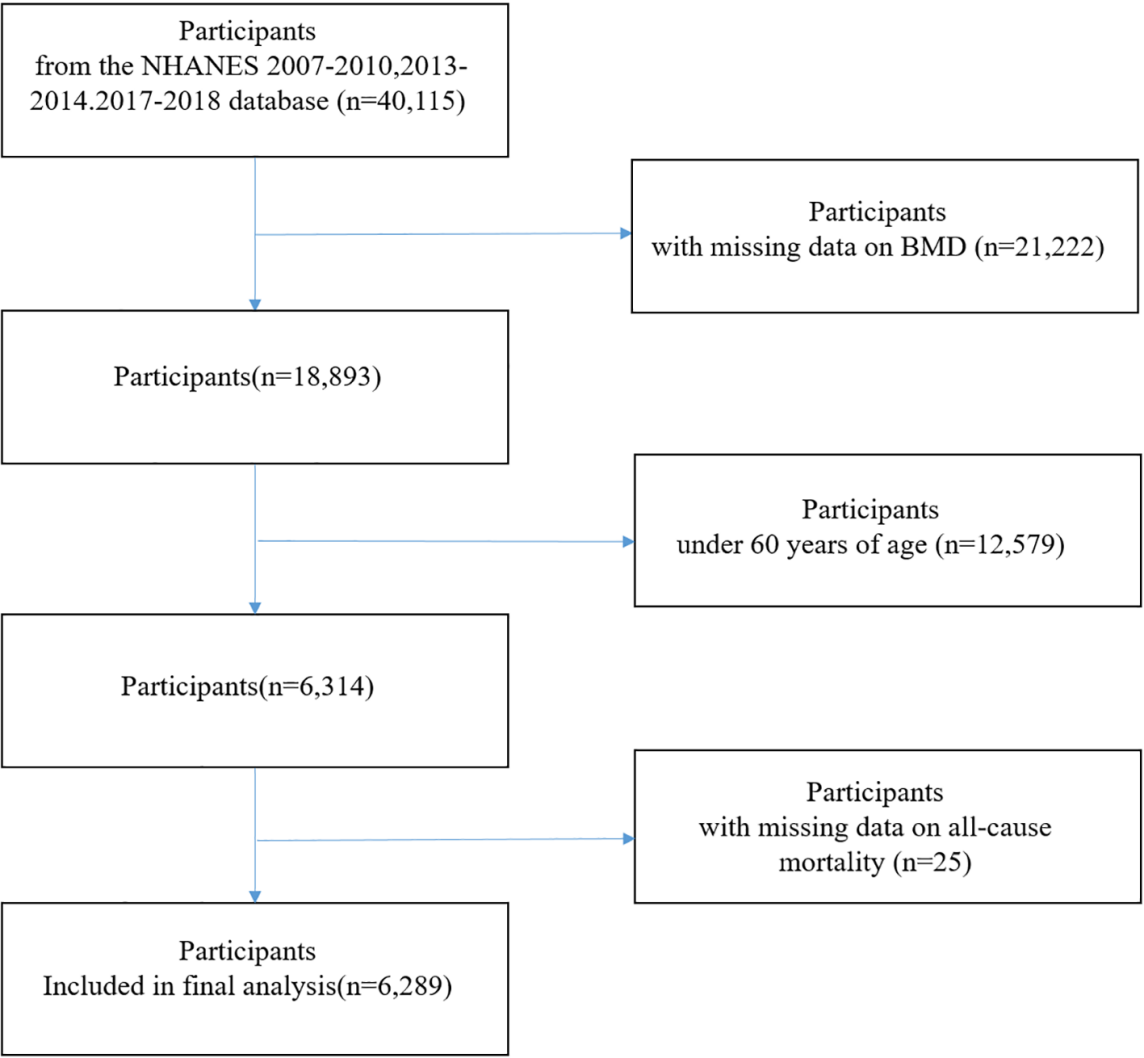
Flow chart of study participants.

For this study, data from five NHANES cycles (2007–2010, 2013–2014, and 2017–2018) were analyzed, along with publicly available linked mortality data through December 31, 2019. NHANES cycles 2011–2012 and 2015–2016 were excluded because BMD measurements via dual-energy X-ray absorptiometry (DXA) were not conducted during these survey periods. Participants aged ≥60 years with complete data on BMD, and mortality status were included. After applying these criteria, a total of 6,289 participants were included in the final analysis (**Figure 1**).

### BMD assessment

BMD was measured using dual-energy X-ray absorptiometry (DXA) with Hologic QDR-4500A fan-beam densitometers (Hologic Inc., Bedford, MA, USA). Measurements were taken at multiple skeletal sites, including total femur, femoral neck, trochanter, and intertrochanter. The left hip was the default site of measurement, but the right hip was used when the left hip was unavailable due to replacement or metal objects.

### Mortality ascertainment

Mortality status was determined by linking NHANES participants to the National Death Index (NDI) using probabilistic record matching. Vital status and cause of death were ascertained using International Classification of Diseases, 10th Revision (ICD-10) codes. The primary outcome of the study was all-cause mortality. Deaths were further classified into three major categories based on the underlying cause of death recorded using ICD-10 codes: cardiovascular disease (CVD) mortality (I00–I09, I11, I13, I20–I51, I60–I69), cancer mortality (C00–C97), and non-cancer non-cardiovascular mortality (all other causes).

### Assessment of covariates

Demographic, socioeconomic, and health-related factors were collected through standardized questionnaires and physical examinations. Covariates included age, gender, race/ethnicity (non-Hispanic White, non-Hispanic Black, Mexican American, Other Hispanic, Other/multiracial and others), education level (less than high school and high school, above high school), smoking status (no, yes), BMI, Serum 25-hydroxyvitamine[25(OH)D), measured by a standardized liquid chromatography-tandem mass spectrometry(LC-MS/MS)method, diabetes, hypertension. The family poverty income ratio (PIR) was determined by dividing the mean income of a family by the poverty threshold specified by the U.S. Department of Health and Human Services.

### Statistical analysis

The data were presented in the form of descriptive statistics, which included means ± standard deviations (SD) for continuous variables and frequencies (percentages) for categorical variables. Differences between groups were assessed using Chi-square tests for categorical variables and linear regression models for continuous variables.

Cox proportional hazards regression models were used to estimate hazard ratios (HRs) and 95% confidence intervals (CIs) for the association between BMD and mortality outcomes. Proportional hazards assumptions were tested and satisfied. Models were adjusted for potential confounders, including demographic, socioeconomic, and clinical variables. Restricted cubic splines were used to evaluate non-linear associations between BMD and mortality risk. Mediation analysis was performed to quantify the indirect effects of BMI on the association between BMD and all-cause mortality. Statistical significance was defined as a two-sided P-value <0.05. The analyses were conducted utilizing R software 3.4.3 and EmpowerStats4.0.

## Results

### Baseline characteristics of study participants

A total of 6,289 participants were included in this study, with 4,867 (77.39%)in the alive group and 1,422(22.61%) in the deceased group. The deceased group was significantly older than the alive group (73.99 ± 6.51 *vs* 68.55 ± 6.52 years, standardized difference(SD) = 0.83, P < 0.001). Regarding gender distribution, the deceased group had a higher proportion of males (59.07% *vs* 49.25%, SD= 0.20, P < 0.001). Non-Hispanic White constituted the largest ethnic group, with a significantly higher proportion in the deceased group (63.92% *vs* 46.74%, SD = 0.39, P < 0.001). Educational attainment analysis showed a higher proportion of participants with high school education or below in the deceased group (60.97% *vs* 50.66%, SD= 0.21, P < 0.001). Additionally, the deceased group demonstrated higher prevalence of smoking (60.21% *vs* 48.16%, SD= 0.24, P < 0.001), diabetes (26.06% *vs* 21.52%, SD= 0.11, P < 0.001), and hypertension (66.57% *vs* 57.93%, SD= 0.18, P < 0.001) (**Table 1**).

**Table 1:** Baseline characteristics of study participants.

all-cause mortality	Total	Alive	Deceased	SD* (95%CI)	P-value
N	6289	4867	1422		
Age (years, mean ± SD)	69.78 ± 6.91	68.55 ± 6.52	73.99 ± 6.51	0.83 (0.77, 0.90)	<0.001
Gender(n,%)				0.20 (0.14, 0.26)	<0.001
Female	3052 (48.53%)	2470 (50.75%)	582 (40.93%)		
Male	3237 (51.47%)	2397 (49.25%)	840 (59.07%)		
Race/ethnicity(n,%)				0.39 (0.33, 0.45)	<0.001
Mexican American	768 (12.21%)	650 (13.36%)	118 (8.30%)		
Non-Hispanic Black	1238 (19.69%)	987 (20.28%)	251 (17.65%)		
Non-Hispanic White	3184 (50.63%)	2275 (46.74%)	909 (63.92%)		
Other Hispanic	594 (9.45%)	506 (10.40%)	88 (6.19%)		
Other/multiracial	505 (8.03%)	449 (9.23%)	56 (3.94%)		
Education level (n,%)				0.21 (0.15, 0.27)	<0.001
Less than high school or high school	3325 (52.99%)	2461 (50.66%)	864 (60.97%)		
Above high school	2950 (47.01%)	2397 (49.34%)	553 (39.03%)		
Smoking status (n,%)				0.24 (0.18, 0.30)	<0.001
No	3087 (49.12%)	2522 (51.84%)	565 (39.79%)		
Yes	3198 (50.88%)	2343 (48.16%)	855 (60.21%)		
Family income to poverty ratio	2.59 ± 1.55	2.70 ± 1.58	2.23 ± 1.38	0.32 (0.26, 0.38)	<0.001
BMI	28.52 ± 5.54	28.78 ± 5.51	27.63 ± 5.55	0.21 (0.15, 0.27)	<0.001
Serum 25(OH)D concentrations(ng/mL, mean ± SD)	73.12 ± 30.46	74.39 ± 30.78	68.52 ± 28.80	0.20 (0.14, 0.26)	<0.001
Waist circumference(cm, mean ± SD)	101.10 ± 13.74	101.13 ± 13.44	100.98 ± 14.74	0.01 (-0.05, 0.07)	0.712
Diabetes (n,%)				0.11 (0.05, 0.17)	<0.001
No	4869 (77.46%)	3819 (78.48%)	1050 (73.94%)		
Yes	1417 (22.54%)	1047 (21.52%)	370 (26.06%)		
Hypertension (n,%)				0.18 (0.12, 0.24)	<0.001
No	2518 (40.11%)	2043 (42.07%)	475 (33.43%)		
Yes	3759 (59.89%)	2813 (57.93%)	946 (66.57%)		
Total femur BMD(g/cm², mean ± SD)	0.91 ± 0.17	0.92 ± 0.16	0.87 ± 0.18	0.29 (0.23, 0.35)	<0.001
Femur neck BMD(g/cm², mean ± SD)	0.75 ± 0.14	0.75 ± 0.14	0.72 ± 0.15	0.25 (0.19, 0.31)	<0.001
Trochanter BMD(g/cm², mean ± SD)	0.69 ± 0.14	0.70 ± 0.14	0.66 ± 0.15	0.25 (0.19, 0.31)	<0.001
Intertrochanter BMD(g/cm², mean ± SD)	1.08 ± 0.20	1.09 ± 0.19	1.03 ± 0.21	0.31 (0.25, 0.37)	<0.001

N, numbers of subjects; %, weighted proportion; CI, confidence interval; SD, standard deviation, SD*, standardized difference; BMI, body mass index; BMD, bone mineral density.

Among the 6,289 participants, the amount of missing values for the covariates were 14 (0.22%) for education level, 634 (10.08%) for family income to poverty ratio, 45 (0.72%) for body mass index, 94 (1.49%) for waist circumference, 381 (6.06%) for serum 25(OH)D concentrations, 12 (0.19%) for hypertension status, 3 (0.05%) for diabetes status, and 4 (0.06%) for smoking status.

### Associations of BMD and other baseline characteristics with all-cause mortality risk

In this prospective cohort study of 6,289 participants, significant inverse associations were observed between BMD measurements and all-cause mortality. All BMD sites were associated with reduced mortality risk: total femur BMD (HR = 0.18, 95% CI: 0.13-0.25), femoral neck BMD (HR = 0.12, 95% CI: 0.08-0.18), trochanter BMD (HR = 0.16, 95% CI: 0.11-0.24), and intertrochanter BMD (HR = 0.23, 95% CI: 0.18-0.30) (all P < 0.0001). When categorized by tertiles, both middle and high BMD groups exhibited significantly reduced mortality risks in comparison to the low BMD group across all sites (HRs ranging from 0.55 to 0.68, all P < 0.0001). Kaplan-Meier analysis demonstrated that participants with low BMD exhibited significantly higher cumulative hazard of all-cause mortality compared to those with middle and high BMD levels during the 160-month follow-up period (p < 0.0001), suggesting a robust association between bone mineral density and mortality risk in older adults (**Supplementary Figure 1**).

Furthermore, an association between age and increased mortality risk was observed (HR = 1.12, 95% CI: 1.11-1.12, P < 0.0001), with elevated risks being observed in the middle and highest age tertiles (HRs = 1.74 and 4.81, respectively). Furthermore, males exhibited a mortality risk that was higher than that of females (HR = 1.47, 95% CI: 1.33-1.64). Non-Hispanic Whites (HR = 1.94, 95% CI: 1.60-2.35) and Non-Hispanic Blacks (HR = 1.57, 95% CI: 1.26-1.96) showed increased risks compared to Mexican Americans. Higher education levels showed protective effects (HR = 0.77, 95% CI: 0.69-0.85).

In terms of lifestyle and health factors, smoking was associated with increased mortality risk (HR = 1.51, 95% CI: 1.36-1.68), while higher BMI was associated with reduced mortality risk (HR = 0.96, 95% CI: 0.95-0.97). When the study population was stratified by tertiles, both middle and high BMI groups exhibited a reduced mortality risk (both HR = 0.69, 95% CI: 0.61-0.78) in comparison to the low BMI group. For waist circumference, only the middle tertile demonstrated a protective effect (HR = 0.83, 95% CI: 0.73-0.94). Conversely, both diabetes and hypertension were found to be associated with increased mortality risk (both HR = 1.42, P < 0.0001). Nevertheless, serum concentrations of 25(OH)D exhibited no substantial correlation with mortality risk (**Table 2**).

**Table 2:** Associations of BMD and other baseline characteristics with all-cause mortality.

Exposure	Statistics	all-cause mortality
Total femur BMD (g/cm², mean ± SD)	0.91 ± 0.17	0.18 (0.13, 0.25) <0.0001
Low	2085 (33.15%)	1.0
Middle	2102 (33.42%)	0.65 (0.57, 0.73) <0.0001
High	2102 (33.42%)	0.56 (0.50, 0.64) <0.0001
Femur neck BMD (g/cm², mean ± SD)	0.75 ± 0.14	0.12 (0.08, 0.18) <0.0001
Low	2080 (33.07%)	1.0
Middle	2101 (33.41%)	0.64 (0.57, 0.73) <0.0001
High	2108 (33.52%)	0.55 (0.48, 0.62) <0.0001
Trochanter BMD (g/cm², mean ± SD)	0.69 ± 0.14	0.16 (0.11, 0.24) <0.0001
Low	2086 (33.17%)	1.0
Middle	2089 (33.22%)	0.61 (0.54, 0.69) <0.0001
High	2114 (33.61%)	0.60 (0.53, 0.68) <0.0001
Intertrochanter BMD (g/cm², mean ± SD)	1.08 ± 0.20	0.23 (0.18, 0.30) <0.0001
Low	2088 (33.20%)	1.0
Middle	2102 (33.42%)	0.68 (0.60, 0.77) <0.0001
High	2099 (33.38%)	0.57 (0.50, 0.65) <0.0001
Age (years, mean ± SD)	69.78 ± 6.91	1.12 (1.11, 1.12) <0.0001
Low	1913 (30.42%)	1.0
Middle	2079 (33.06%)	1.74 (1.46, 2.09) <0.0001
High	2297 (36.52%)	4.81 (4.10, 5.64) <0.0001
Gender (n,%)		
Female	3052 (48.53%)	1.0
Male	3237 (51.47%)	1.47 (1.33, 1.64) <0.0001
Race/ethnicity (n,%)		
Mexican American	768 (12.21%)	1.0
Non-Hispanic Black	1238 (19.69%)	1.57 (1.26, 1.96) <0.0001
Non-Hispanic White	3184 (50.63%)	1.94 (1.60, 2.35) <0.0001
Other Hispanic	594 (9.45%)	0.99 (0.75, 1.30) 0.9239
Other/multiracial	505 (8.03%)	1.22 (0.89, 1.67) 0.2264
Education level (n,%)		
Less than high school or high school	3325 (52.99%)	1.0
Above high school	2950 (47.01%)	0.77 (0.69, 0.85) <0.0001
Smoking status (n,%)		
No	3087 (49.12%)	1.0
Yes	3198 (50.88%)	1.51 (1.36, 1.68) <0.0001
BMI (mean ± SD)	28.52 ± 5.54	0.96 (0.95, 0.97) <0.0001
Low	2080 (33.31%)	1.0
Middle	2067 (33.10%)	0.69 (0.61, 0.78) <0.0001
High	2097 (33.58%)	0.69 (0.61, 0.78) <0.0001
Waist circumference (cm, mean ± SD)	101.10 ± 13.74	1.00 (1.00, 1.00) 0.9732
Low	2055 (33.17%)	1.0
Middle	2059 (33.24%)	0.83 (0.73, 0.94) 0.0048
High	2081 (33.59%)	1.00 (0.88, 1.13) 0.9902
Serum 25 (OH)D concentrations(ng/mL, mean ± SD)	73.12 ± 30.46	1.00 (1.00, 1.00) 0.0766
Low	1965 (33.26%)	1.0
Middle	1972 (33.38%)	0.91 (0.80, 1.04) 0.1605
High	1971 (33.36%)	0.91 (0.79, 1.04) 0.1765
Hypertension (n,%)		
No	2518 (40.11%)	1.0
Yes	3759 (59.89%)	1.42 (1.27, 1.59) <0.0001
Diabetes (n,%)		
No	4869 (77.46%)	1.0
Yes	1417 (22.54%)	1.42 (1.26, 1.60) <0.0001

N, numbers of subjects; %, weighted proportion; SD, standard deviation, BMI, body mass index; BMD, bone mineral density.

### Multivariate cox regression analysis of BMD and all-cause mortality

We employed Cox proportional hazards models to evaluate the association between BMD at different sites and all-cause mortality risk. In the unadjusted model, BMD at all measurement sites showed significant negative associations with mortality risk. These associations persisted after adjusting for demographic characteristics (age, gender, race, education, and poverty-income ratio) in Model I. After further adjustment for clinical characteristics (BMI, waist circumference, vitamin D levels, hypertension, and diabetes) in Model II, all BMD indicators maintained their significant protective effects. Specifically, in the fully adjusted model, total femur BMD (HR = 0.19, 95% CI: 0.12-0.30, P < 0.0001), femoral neck BMD (HR = 0.26, 95% CI: 0.15-0.43, P < 0.0001), trochanter BMD (HR = 0.17, 95% CI: 0.11-0.29, P < 0.0001), and intertrochanter BMD (HR = 0.26 95% CI: 0.17-0.38, P < 0.0001) were all significantly associated with reduced mortality risk (**Table 3**). These associations remained robust after adjustment for potential confounding factors.

**Table 3:** Associations between BMD and all-cause mortality using different adjustment models.

Exposure	Non-adjusted	Adjust I	Adjust II
Total femur BMD	0.18 (0.13, 0.25) <0.0001	0.15 (0.10, 0.23) <0.0001	0.19 (0.12, 0.30) <0.0001
Femur neck BMD	0.12 (0.08, 0.18) <0.0001	0.19 (0.12, 0.30) <0.0001	0.26 (0.15, 0.43) <0.0001
Trochanter BMD	0.16 (0.11, 0.24) <0.0001	0.12 (0.08, 0.19) <0.0001	0.17 (0.11, 0.29) <0.0001
Intertrochanter BMD	0.23 (0.18, 0.30) <0.0001	0.22 (0.16, 0.31) <0.0001	0.26 (0.17, 0.38) <0.0001

Adjust I model adjust for: Age; Gender; Race/ethnicity; Education level; Family income to poverty ratio.

Adjust II model adjust for: Age; Gender; Race/ethnicity; Education level; Family income to poverty ratio; Body mass index; Waist circumference; Serum 25(OH)D concentrations; Hypertension; Diabetes; Smoking status.

To further explore the underlying mechanisms, we conducted cause-specific mortality analyses, which revealed that BMD demonstrated protective associations across all death causes examined. The cause-specific mortality analysis showed that BMD had the strongest protective effect against cardiovascular disease (CVD) mortality, with hazard ratios ranging from 0.11 to 0.18 across different skeletal sites after full adjustment. The associations with cancer mortality were weaker but remained significant (HRs 0.19-0.29), while non-cancer non-CVD mortality showed intermediate protective effects (HRs 0.18-0.35). These results suggest that the bone-mortality relationship varies by underlying disease mechanisms, with cardiovascular pathways being most prominently involved (**Supplementary Table 1**).

### Threshold effects of BMD on mortality risk

Piecewise Cox regression models were used to investigate the non-linear relationship between BMD and all-cause mortality. Model I, which assumed a linear relationship, showed that BMD at all four sites was significantly associated with lower all-cause mortality. However, Model II revealed significant non-linear relationships between BMD and mortality (logarithmic likelihood ratio test P<0.001 for all sites). For total femur BMD, the threshold was 0.65 g/cm². Below this threshold, the mortality risk was significantly reduced (HR = 0.00, 95% CI: 0.00-0.01, P < 0.0001); above the threshold, a protective effect remained, but was attenuated (HR = 0.27, 95% CI: 0.16-0.43, P < 0.0001). Similar non-linear patterns were observed for femoral neck BMD (threshold 0.66 g/cm²) and trochanter BMD (threshold 0.68 g/cm²), with significant protective effects below the thresholds and non-significant associations above. For intertrochanter BMD (threshold 0.77 g/cm²), protective effects were observed both below and above the threshold, but with differing magnitudes of effect (**Table 4**).

**Table 4:** Threshold effects of BMD on all-cause mortality using piecewise cox regression analysis.

Parameters	Total femur BMD OR (95% CI) *P*	Femur neck BMD OR (95% CI) *P*	Trochanter BMD OR (95% CI) *P*	Intertrochanter BMD OR (95% CI) *P*
Model I^b^				
One-line slope	0.19 (0.12, 0.30) <0.0001	0.26 (0.15, 0.43) <0.0001	0.17 (0.11, 0.29) <0.0001	0.26 (0.17, 0.38) <0.0001
Model II^c^				
Inflection point (K), °C	0.65	0.66	0.68	0.77
<K	0.00 (0.00, 0.01) <0.0001	0.02 (0.00, 0.06) <0.0001	0.04 (0.02, 0.11) <0.0001	0.01 (0.00, 0.04) <0.0001
>K	0.27 (0.16, 0.43) <0.0001	0.67 (0.35, 1.26) 0.2141	0.54 (0.25, 1.15) 0.1115	0.34 (0.23, 0.52) <0.0001
Slope 2 – Slope 1	187.92 (20.88, 1691.59) <0.0001	40.31 (8.66, 187.70) <0.0001	12.21 (3.27, 45.64) 0.0002	49.01 (8.58, 279.86) <0.0001
LRT^d^	<0.001	<0.001	<0.001	<0.001

a Adjusted variables: Age; Gender; Race/ethnicity; Education level; Family income to poverty ratio; Body mass index; Waist circumference; Serum 25(OH)D concentrations; Hypertension; Diabetes; Smoking status.

b Linear analysis, P-value <0.05 indicates a linear relationship.

c Non-linear analysis.

d P-value <0.05 means Model II is significantly different from Model I, which indicates a non-linear relationship.

OR, odds ratio; CI, confidence interval; LRT, logarithmic likelihood ratio test.

To further characterize the threshold effects across different mortality outcomes, we applied the same piecewise Cox regression analysis to cause-specific mortality. The threshold analysis demonstrated that BMD exhibited distinct protective patterns below and above the inflection points for different death causes. For CVD mortality, the threshold values ranged from 0.50 to 0.77 g/cm² across skeletal sites, with hazard ratios below thresholds ranging from 0.00 to 0.06 and above thresholds from 0.16 to 0.41. Cancer mortality showed higher threshold values (0.62-1.01 g/cm²) with less pronounced threshold effects, while non-cancer non-cardiovascular mortality displayed intermediate patterns (thresholds 0.65-0.77 g/cm²). These findings suggest that the threshold effects of BMD on mortality risk are disease-specific, with cardiovascular outcomes showing the most pronounced non-linear relationships (**Supplementary Table 2**).

### Non-linear dose-response relationship between BMD and all-cause mortality

**Figure 2:**
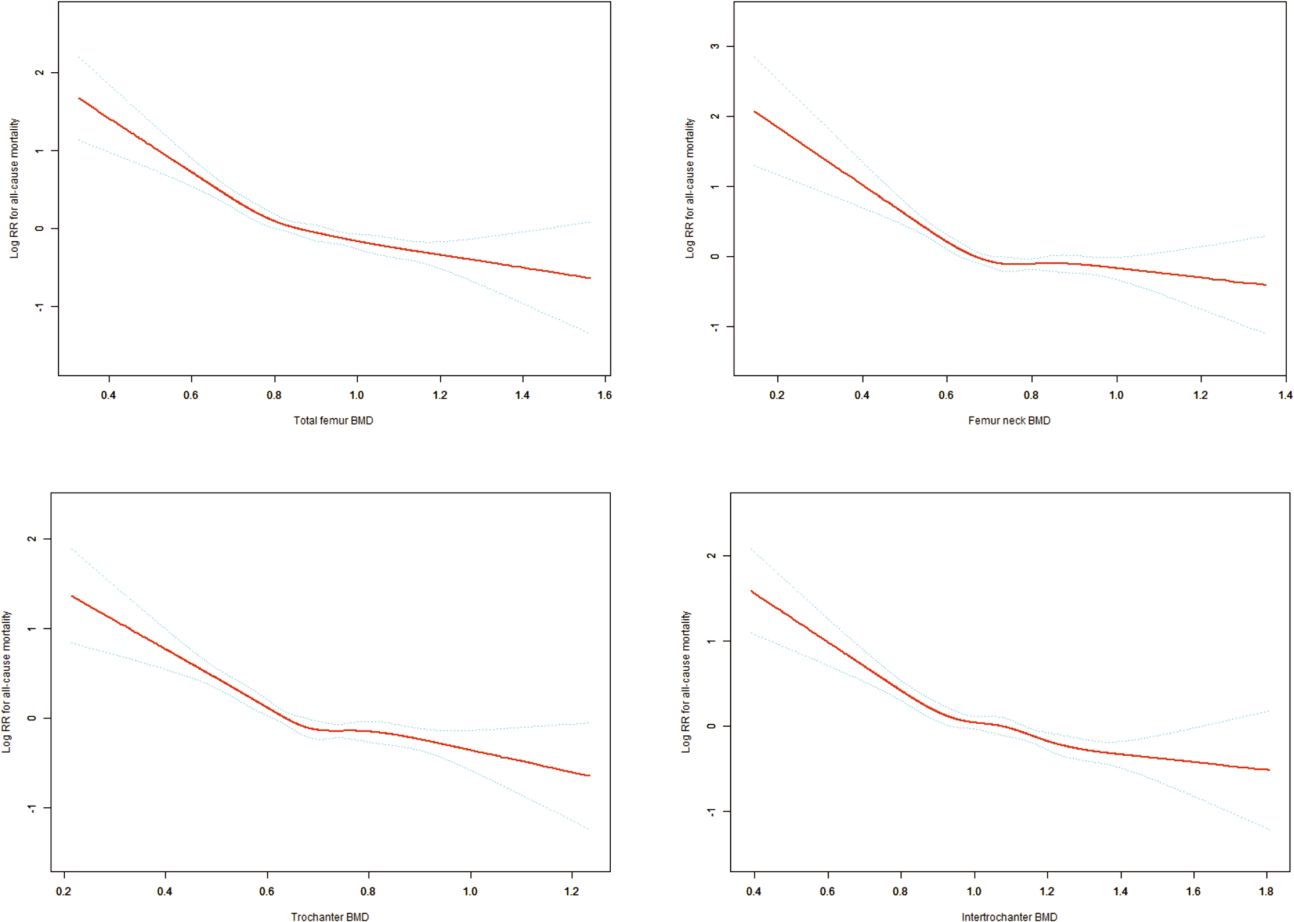
Non-linear Dose-Response Relationships Between Hip BMD Measurements and All-Cause Mortality. Adjusting variables: Age; Gender; Race/ethnicity; Education level; Family income to poverty ratio; Body mass index; Waist circumference; Serum 25(OH)D concentrations; Hypertension; Diabetes; Smoking status.

Restricted cubic spline analysis revealed significant non-linear relationships between BMD at all four sites (total femur, femoral neck, trochanter, and intertrochanter) and the log relative risk of all-cause mortality. All sites demonstrated similar patterns: a steep decline in mortality risk with increasing BMD at lower values, followed by a plateauing effect after reaching specific thresholds. Specifically, the curve slopes changed notably around 0.65 g/cm² for total femur BMD, 0.66 g/cm² for femoral neck BMD, 0.68 g/cm² for trochanter BMD, and 0.77 g/cm² for intertrochanter BMD. The 95% confidence intervals were relatively narrow in the middle range but widened at extreme values for all curves, indicating greater uncertainty in estimates at these extremes. This non-linear relationship suggests a threshold effect, where the protective effect of increasing BMD on mortality risk diminishes beyond certain thresholds (**Figure 2**).

### Non-linear association between BMI and all-cause mortality

In older adults (aged ≥60 years), BMI exhibited a non-linear relationship with all-cause mortality, demonstrating a significant threshold effect at 27.43. Below this threshold (BMI < 27.43), a 16% reduction in mortality risk was observed for each unit increase in BMI (HR = 0.84, 95% CI: 0.81-0.88, P < 0.0001). In the population with a BMI above the threshold (BMI > 27.43), the protective effect was weaker but still significant, with each unit increase associated with a 4% reduction in mortality risk (HR = 0.96, 95% CI: 0.94-0.99, P = 0.01) (**Table 5**) (**Supplementary Figure 2**). These associations remained robust after adjusting for demographic characteristics, socioeconomic status, and clinical factors.

**Table 5:** Threshold effect analysis of BMI on all-cause mortality in older adults.

Parameters	All-cause mortality OR (95% CI)	P-value
Model I^b^		
One-line slope	0.93 (0.90, 0.95)	<0.0001
Model II^c^		
Inflection point (K), °C	27.43	
<K	0.84 (0.81, 0.88)	<0.0001
>K	0.96 (0.94, 0.99)	0.01
Slope 2 – Slope 1	1.14 (1.10, 1.19)	<0.0001
LRT^d^	<0.001	

a Adjusted variables: Age; Gender; Race/ethnicity; Education level; Family income to poverty ratio; Waist circumference; Serum 25(OH)D concentrations; Hypertension; Diabetes; Smoking status.

b Linear analysis, P-value <0.05 indicates a linear relationship.

c Non-linear analysis.

d P-value <0.05 means Model II is significantly different from Model I, which indicates a non-linear relationship.

OR, odds ratio; CI, confidence interval; LRT, logarithmic likelihood ratio test.

### BMI-mediated effects in BMD-mortality association

**Figure 3:**
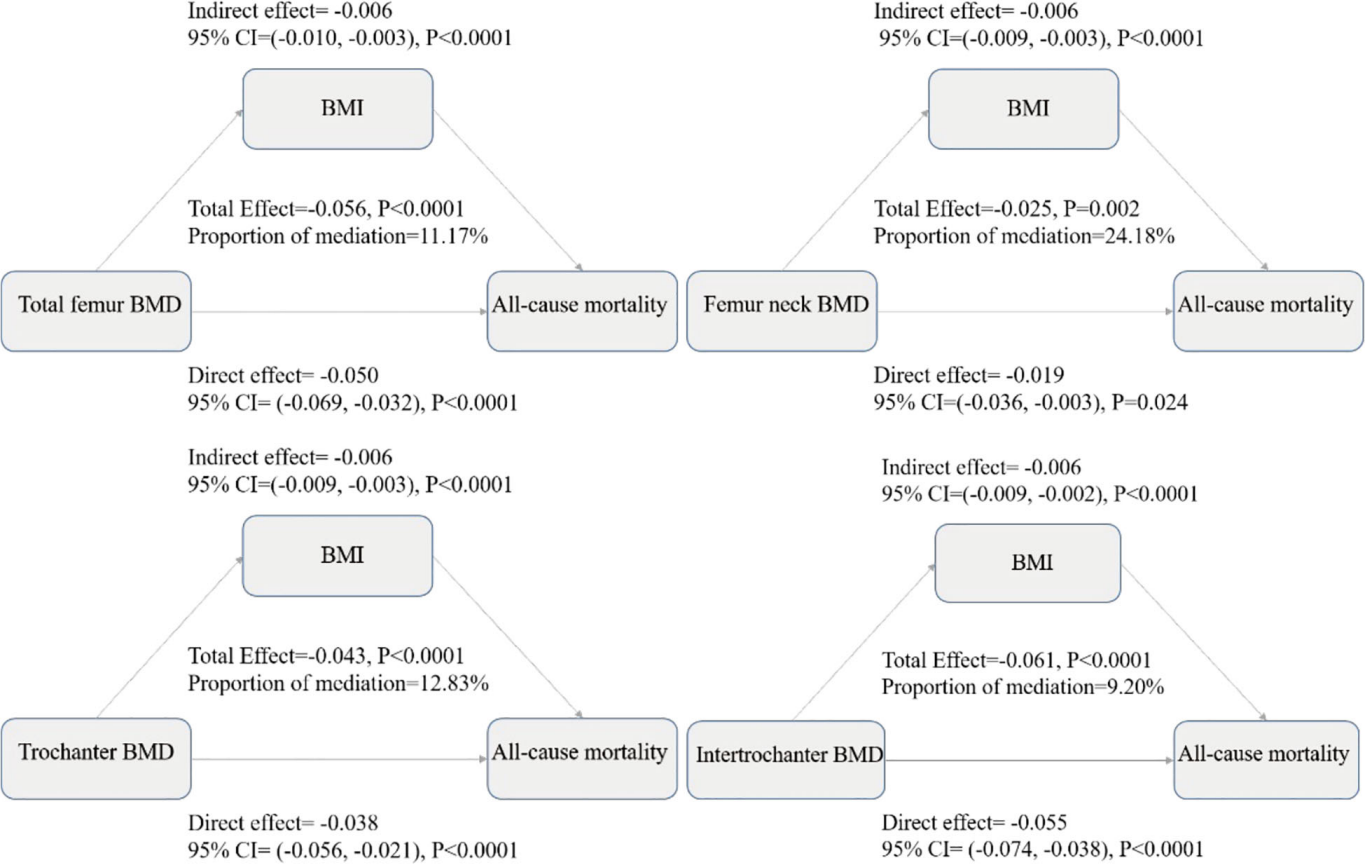
Mediation analysis decomposes the total observed BMD-mortality association into direct and indirect components, where the total effect represents the overall association between BMD and mortality risk, the direct effect represents the BMD-mortality association independent of BMI, the indirect effect represents the portion of association that may operate through BMI as an intermediate variable, and the proportion of mediation indicates the percentage of total effect that may be explained by BMI-related pathways. All effects are presented as coefficients with 95% confidence intervals from multiple-adjusted models including age, gender, race/ethnicity, education level, family income to poverty ratio, body mass index, waist circumference, serum 25(OH)D concentrations, hypertension, diabetes, and smoking status. Negative values indicate associations with reduced mortality risk, and the significant direct and indirect effects observed across all sites suggest that BMD may be associated with mortality risk through both BMI-related and BMI-independent pathways in this observational analysis.

To explore potential pathways underlying the observed BMD-mortality associations, we conducted mediation analysis examining whether BMI appeared to serve as an intermediary variable in these relationships. Mediation analysis decomposes the total observed association into two components (1): direct effects representing the BMD-mortality association independent of BMI, and (2) indirect effects representing the portion of association that may operate through BMI as an intermediate variable. **Figure 3** presents the comprehensive mediation analysis results across four hip BMD sites. The analysis revealed that BMI appeared to partially mediate the associations between BMD and all-cause mortality, after adjusting for demographic characteristics (age, gender, race), socioeconomic status (education, poverty-income ratio), and clinical factors (smoking status, diabetes, hypertension, vitamin D levels, and waist circumference). The total effects (representing the overall BMD-mortality associations) were strongest for total femur BMD (-0.056, P<0.0001), followed by intertrochanter (-0.061, P<0.0001), trochanter (-0.043, P<0.0001), and femoral neck (-0.025, P=0.002), with negative coefficients indicating associations with reduced mortality risk. The mediation analysis showed that part of these associations appeared to operate through BMI-related pathways. The proportion of the total effect that may be explained by BMI-mediated pathways (shown as “Proportion of mediation” in **Figure 3**) varied across anatomical sites: femoral neck showed the highest mediation proportion (24.18%, 95% CI: 9.60%-67.66%), suggesting that nearly one-quarter of the BMD-mortality association at this site may be related to BMI pathways. This was followed by trochanter (12.83%, 95% CI: 5.51%-24.22%), total femur (11.17%, 95% CI: 4.45%-19.55%), and intertrochanter (9.20%, 95% CI: 3.44%-15.94%). The significant direct effects observed across all sites (all P<0.0001) suggest that BMD may be associated with mortality risk through multiple pathways beyond BMI alone, while the statistically significant indirect effects (all P<0.0001) suggest that BMI may serve as a meaningful intermediary pathway in the BMD-mortality relationships.

## Discussion

In this extensive cohort study, which encompassed a total of 6,289 participants aged at least 60 years, significant associations were observed between site-specific BMD measurements and all-cause mortality. During the course of the follow-up period, 1,422 deaths (equaling 22.61%) were recorded. The analysis suggested a J-shaped relationship between BMD and mortality risk. The protective effect against mortality was most pronounced for total femur BMD (total effect: -0.056, P<0.0001), followed by intertrochanter (-0.061, P<0.0001), trochanter (-0.043, P<0.0001), and femoral neck (-0.025, P=0.002). It is noteworthy that BMI appeared to have a substantial mediating role in this association, with the extent of mediation varying by anatomical location (9.20%-24.18%). These associations remained robust after thorough adjustment for potential confounders.

In the present analysis, potential confounding factors were taken into consideration, including demographic characteristics (age, gender, race/ethnicity) and socioeconomic indicators (education, income). These factors have been identified as established determinants of bone health and mortality. It is also worthy of note that we adjusted for serum 25(OH)D concentrations, given the crucial role of vitamin D in bone metabolism and its independent associations with mortality through effects on immune function and cardiovascular health (6). Additional adjustments included anthropometric measures (BMI, waist circumference), comorbidities (hypertension, diabetes), and smoking status.

In their study, Shi et al. investigated the associations between BMD and long-term risks of cardiovascular disease, cancer, and all-cause mortality using data from NHANES III (1988–1994) with follow-up until December 31, 2015. Amongst the 11,264 participants aged 18 and above (467 with osteoporosis, 4,113 with osteopenia, and 6,684 with normal bone mass), the study found that osteopenia and osteoporosis were independently associated with an increased risk of all-cause mortality (hazard ratio [HR] = 1.37 and HR = 1.06, respectively), with stronger associations observed in older participants and those with a lower BMI (7).A large-scale cohort study utilized NHANES data (2005–2010, 2013–2014) to examine the relationship between BMD and mortality in 15,076 U.S. adults (mean age 48.6 years). Utilising DXA measurements at multiple skeletal sites and a median 6.8-year follow-up period, the study established that osteoporosis was associated with elevated all-cause mortality risk in the total femur (HR=1.36), femur neck (HR=1.41), and intertrochanter (HR=1.34) regions. The study revealed an L-shaped relationship between BMD and mortality, with gender-specific effects: higher BMD levels were more protective against cancer mortality in males and against heart disease mortality in females (8). Our study focused on older adults (≥60 years), a demographic particularly susceptible to age-related bone loss, characterized by significant changes in bone metabolism and skeletal homeostasis including decreased bone formation, increased bone resorption, and altered calcium homeostasis. Rather than categorizing participants based on osteoporosis status, we investigated the continuous relationship between BMD and mortality to better understand the dose-response effects. Our analysis revealed threshold effects for BMD measurements at different femoral sites: total femur (0.65 g/cm²), femoral neck (0.66 g/cm²), trochanter (0.68 g/cm²), and intertrochanter (0.77 g/cm²). Notably, these identified thresholds closely approximate the established diagnostic cutoff points for osteoporosis (total femur: 0.67 g/cm², femoral neck: 0.56 g/cm², trochanter: 0.46 g/cm², intertrochanter: 0.79 g/cm²), suggesting a clinically relevant transition point in the BMD-mortality relationship.

To contextualize these threshold values within established clinical frameworks, it is important to compare them with WHO diagnostic criteria. According to the 2023 International Society for Clinical Densitometry (ISCD) official positions, the WHO international reference standard for osteoporosis diagnosis is a T-score of -2.5 or less at the femoral neck, with the reference standard calculated from the female, white, age 20–29 years, NHANES III database (9). Based on this established reference standard, our identified thresholds correspond to approximate T-scores of: femoral neck (0.66 g/cm²) ≈ T-score -1.65, total femur (0.65 g/cm²) ≈ T-score -2.58, trochanter (0.68 g/cm²) ≈ T-score -2.33, and intertrochanter (0.77 g/cm²) ≈ T-score -1.58. These values largely fall within the osteopenic range (T-scores between -1.0 and -2.5), with some approaching the osteoporosis diagnostic threshold (T-score ≤ -2.5) (10).We utilized absolute BMD values rather than T-scores in our analysis to allow for more precise threshold identification in piecewise regression models and to avoid potential variability introduced through standardization processes. Our observational findings suggest that the association between BMD and mortality may be most pronounced at bone density levels that are clinically recognized as compromised but not yet severely osteoporotic.

Li et al. studied the BMD-mortality association in 2,102 Type 2 Diabetes Mellitus patients using NHANES data (2005–2010, 2013–2014), finding that osteoporosis and osteopenia independently increased all-cause mortality (HR = 3.33 and 1.14) and CVD mortality risks (11). While both studies used NHANES data, key differences exist: our study revealed a J-shaped BMD-mortality association in older adults versus their linear correlation in T2DM patients, identified site-specific threshold effects, and uniquely quantified BMI’s mediating role in this relationship”.

The cause-specific mortality analyses provide additional mechanistic insights beyond all-cause mortality. Our findings revealed differential associations between BMD and various death causes, with the strongest associations observed for CVD mortality (HRs 0.11-0.18), followed by non-cancer non-cardiovascular mortality (HRs 0.18-0.35), and weaker associations with cancer mortality (HRs 0.19-0.29). The threshold analyses showed disease-specific non-linear patterns, with CVD mortality exhibiting the most pronounced threshold relationships across BMD sites. These observations align with emerging evidence linking bone metabolism and cardiovascular health through shared pathways involving inflammation, oxidative stress, and mineral metabolism (12, 13). The weaker associations with cancer mortality may reflect the heterogeneous nature of malignant diseases. These findings support the clinical relevance of BMD assessment beyond fracture risk evaluation.

The mediation analysis suggests that BMI may serve as an intermediary factor between BMD and all-cause mortality. This suggests that the observed associations between BMD and all-cause mortality may operate partly through pathways involving BMI. Specifically, the observed associations suggest potential relationships between BMD, BMI, and mortality rates. However, it is imperative to acknowledge that this correlation does not establish a direct causal relationship, where an increase in BMD necessarily results in an increase in BMI, nor does it ensure that an increase in BMI will lead to a decrease in all-cause mortality. The relationship is subject to significant modulation by various factors, including individual health status, lifestyle choices, and environmental influences (14). The present findings are in alignment with those previously reported by Sun et al (15)., which demonstrate that modest increases in BMI are associated with a reduced risk of mortality in older adults. Specifically, in our study the present cohort of adults aged ≥60 years, a non-linear relationship between BMI and all-cause mortality was identified, with a significant threshold effect at 27.43. The protective effect was particularly pronounced below this threshold, where each unit increase in BMI corresponded to a 16% reduction in mortality risk (HR = 0.84, 95% CI: 0.81-0.88, P < 0.0001). Conversely, the protective effect above the threshold was observed to be less pronounced, with each unit increase in BMI corresponding to a 4% reduction in mortality risk (HR = 0.96, 95% CI: 0.94-0.99, P = 0.01).

In the elderly population, the interplay between BMI and all-cause mortality becomes increasingly intricate, often described as the “obesity paradox.” The extant research suggests that, in the case of older adults, a moderate degree of overweight or mild obesity may confer health benefits. Conversely, a low BMI has been demonstrated to be associated with elevated mortality risks (16). For instance, research has demonstrated that in elderly subjects, especially those over 85 years of age, the correlation between elevated BMI and mortality rates becomes less pronounced. This finding suggests that maintaining a healthy BMI is crucial for achieving a long lifespan (17). The complex interrelationship between BMI and BMD is characterized by a non-linear relationship, whereby elevated BMI does not necessarily result in improved bone health outcomes (18). It should be noted that BMI as a ‘mediator’ in our analysis represents a statistical concept rather than direct biological causation. BMD and BMI likely represent different manifestations of frailty syndrome, with BMI reflecting nutritional-metabolic status that helps explain part of the mechanisms underlying the BMD-mortality association.

The observed J-shaped association between BMD and mortality reflects complex physiological mechanisms involving hormonal regulation, nutritional factors, and metabolic interactions. The skeletal system undergoes continuous remodeling through balanced osteoblast and osteoclast activity, and BMD decline indicates an imbalance in this process, resulting in increased fragility and heightened fracture risk, which are substantial contributors to mortality in elderly populations (19). Hormonal changes, particularly the decline in sex hormones with age, significantly impact bone metabolism through regulation of osteoblast and osteoclast activity, with estrogen and testosterone deficiency resulting in heightened bone resorption and diminished bone formation (20, 21). Research has demonstrated that sex hormones play crucial roles in bone mineral density regulation, with their age-related decline contributing to increased mortality risk through accelerated bone loss (22). The crosstalk between bone and gonads represents another critical pathway, where bone-derived osteocalcin regulates testosterone production while sex hormones reciprocally influence bone metabolism, creating a bidirectional endocrine loop that becomes increasingly important with aging (23). Our finding that BMI mediates 9.20-24.18% of the BMD-mortality relationship across different anatomical sites suggests that nutritional status and body composition play crucial roles in this association. Higher BMI may confer protection through improved nutritional reserves, enhanced estrogen production from adipose tissue, and increased mechanical loading on bones, while also providing metabolic buffering against the catabolic effects of chronic diseases. The site-specific variations in BMI mediation (femoral neck: 24.18% *vs*. intertrochanter: 9.20%) likely reflect differences in mechanical loading patterns and cortical-trabecular bone composition at these anatomical locations, with the femoral neck representing a critical load-bearing region that is highly sensitive to mechanical loading and metabolic factors related to body mass index (24).

The BMD-mortality relationship extends beyond simple fracture risk, representing broader metabolic dysfunction where alterations in bone metabolism serve as indirect expressions of systemic health deterioration. The role of sarcopenia is particularly significant in explaining our BMI-mediated findings, as sarcopenia and osteoporosis frequently coexist in aging populations, with both conditions associated with increased risk of adverse health outcomes including fractures, dysmobility, and mortality (25). This osteosarcopenia creates a synergistic effect that substantially amplifies mortality risk while potentially masking the protective effects of higher BMI through replacement of metabolically active muscle tissue with adipose tissue. Physical exercise emerges as a fundamental modulator that can enhance both BMD and BMI benefits through mechanical loading, hormonal optimization, and improved muscle-bone interactions (26). The threshold effects we identified suggest that maintaining optimal BMI becomes increasingly important as BMD declines, as metabolic bone diseases negatively impact overall health and quality of life, placing individuals at high risk for fracture and increasing morbidity and mortality (27). These findings emphasize that clinical interventions for older adults with low BMD should adopt integrated approaches targeting both bone density and body mass management, as the protective effects of BMI appear most pronounced in individuals at greatest skeletal risk. However, it is important to note that these observational findings cannot establish causality, and the observed associations may reflect complex underlying relationships rather than direct causal pathways.

### Limitations

It is imperative that the limitations of this study are acknowledged. Firstly, as with any observational study, unmeasured confounding factors might exist despite comprehensive adjustment. Although common risk factors known to be associated with mortality were adjusted for and sensitivity analyses were conducted that showed robust results, the influence of unmeasured confounders cannot be completely ruled out. Secondly, the reliance on NHANES data, while ensuring national representation, is subject to inherent limitations. The self-reported nature of data on household income, education, smoking history, diabetes history and hypertension history may be subject to reporting bias. Additionally, the single time point for BMD measurement may not fully capture the dynamic nature of bone metabolism over time. Thirdly, while the present study concentrated on all-cause mortality, no examination was made of disease-specific mortality outcomes, which might have provided additional insights into the mechanisms linking BMD with mortality. Furthermore, the lack of information about interventions and treatments during the follow-up period might have influenced the relationship between BMD and mortality outcomes.

## Conclusion

This study analyzed data from 6,289 NHANES participants aged ≥60 years and observed a J-shaped association between BMD and all-cause mortality, with site-specific threshold effects at different hip locations. The findings suggested that mortality risk increased as BMD decreased, with stronger protective effects observed below specific threshold values. Additionally, BMI appeared to have significant mediating effects in this relationship, particularly at the femoral neck. The insights derived from this study offer valuable perspectives for the early risk stratification and intervention strategies in older populations, emphasizing the importance of integrated approaches targeting both bone density and body mass management. However, it is important to note that these observational findings cannot establish causality, and the observed associations may reflect complex underlying relationships rather than direct causal pathways.

## Data Availability

Publicly available datasets were analyzed in this study. This data can be found here: The datasets generated and analyzed in the current study are available at the NHANES website (https://www.cdc.gov/Nchs/Nhanes).
