# Supplementary material for: Threshold effects of bone mineral density on mortality risk: a comprehensive analysis of BMI-mediated pathways in older population
Source: Front Endocrinol (Lausanne). 2025 Jul 22;16:1567047. doi: 10.3389/fendo.2025.1567047 (PMC12321544; doi:10.3389/fendo.2025.1567047)
Supplement: Supplementary file 1 [file Image1.pdf]

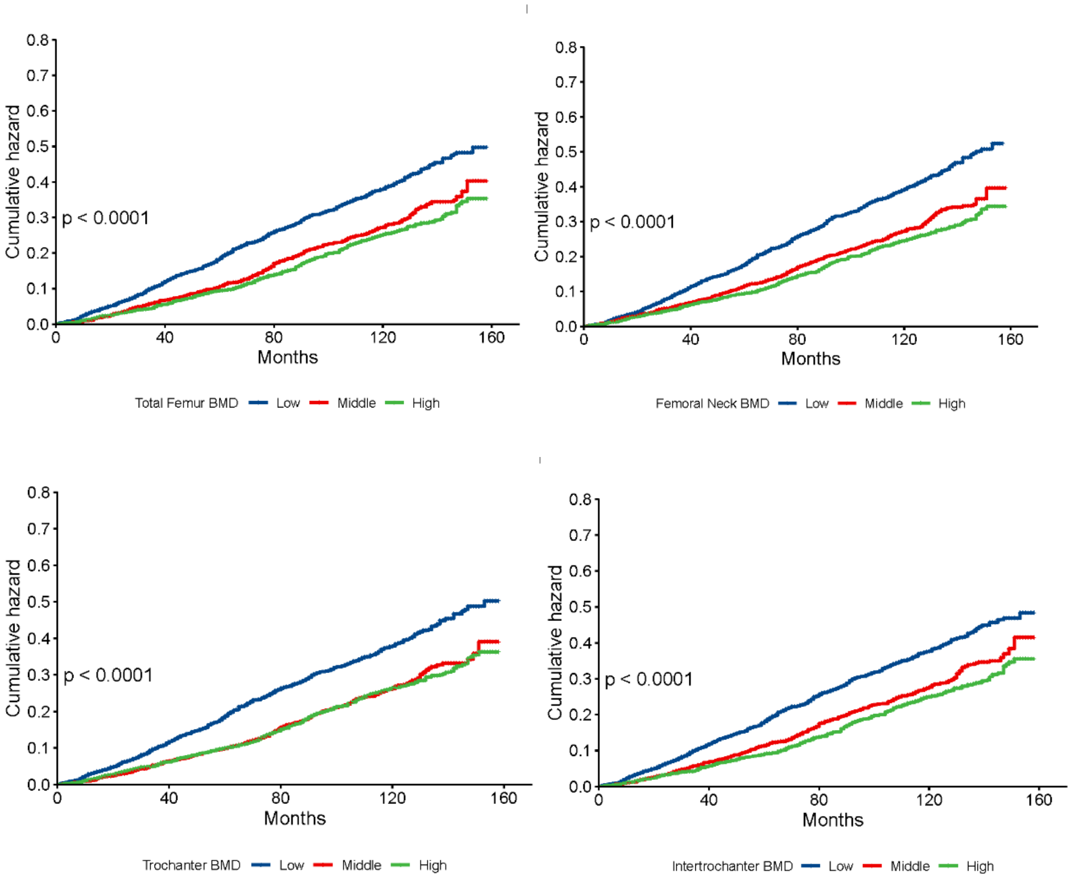

Supplemental Fig.1 Cumulative hazard of all-cause mortality by BMD levels. Kaplan-Meier analysis demonstrated that participants with low BMD exhibited significantly higher cumulative hazard of all-cause mortality compared to those with middle and high BMD levels during the 160-month follow-up period ($p < 0.0001$), suggesting a robust association between bone mineral density and mortality risk in older adults.
