# Supplementary material for: Threshold effects of bone mineral density on mortality risk: a comprehensive analysis of BMI-mediated pathways in older population
Source: Front Endocrinol (Lausanne). 2025 Jul 22;16:1567047. doi: 10.3389/fendo.2025.1567047 (PMC12321544; doi:10.3389/fendo.2025.1567047)
Supplement: Supplementary file 2 [file Image2.pdf]

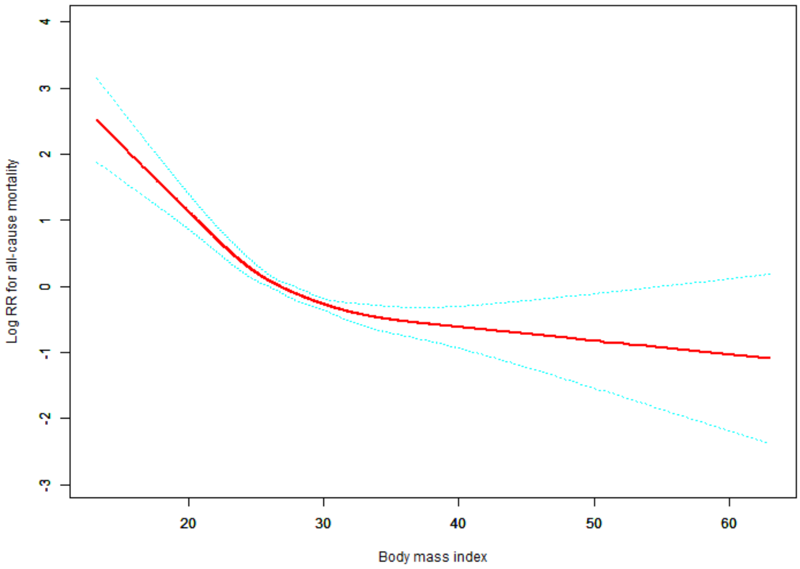

Supplemental Fig.2 Non-linear Association between BMI and All-cause Mortality in Older Adults. Adjusted variables: Age; Gender; Race/ethnicity; Education level; Family income to poverty ratio; Waist circumference; Serum 25(OH)D concentrations; Hypertension; Diabetes; Smoking status. This figure reveals a J-shaped relationship between BMI and mortality risk in older adults.
