# Supplementary material for: Threshold effects of bone mineral density on mortality risk: a comprehensive analysis of BMI-mediated pathways in older population
Source: Front Endocrinol (Lausanne). 2025 Jul 22;16:1567047. doi: 10.3389/fendo.2025.1567047 (PMC12321544; doi:10.3389/fendo.2025.1567047)
Supplement: Supplementary file 3 [file Table1.pdf]

Supplementary Table 1 Association between bone mineral density and cause-specific mortality

Exposure	Non-adjusted	Adjust I	Adjust II
all-cause mortality			
Total femur BMD	0.18 (0.13, 0.25) <0.0001	0.15 (0.10, 0.23) <0.0001	0.19 (0.12, 0.30) <0.0001
Femur neck BMD	0.12 (0.08, 0.18) <0.0001	0.19 (0.12, 0.30) <0.0001	0.26 (0.15, 0.43) <0.0001
Trochanter BMD	0.16 (0.11, 0.24) <0.0001	0.12 (0.08, 0.19) <0.0001	0.17 (0.11, 0.29) <0.0001
Intertrochanter BMD	0.23 (0.18, 0.30) <0.0001	0.22 (0.16, 0.31) <0.0001	0.26 (0.17, 0.38) <0.0001
CVD mortality			
Total femur BMD	0.17 (0.09, 0.33) <0.0001	0.15 (0.07, 0.33) <0.0001	0.12 (0.05, 0.29) <0.0001
Femur neck BMD	0.09 (0.04, 0.21) <0.0001	0.17 (0.07, 0.42) 0.0001	0.15 (0.06, 0.43) 0.0003
Trochanter BMD	0.15 (0.07, 0.33) <0.0001	0.11 (0.04, 0.26) <0.0001	0.11 (0.04, 0.29) <0.0001
Intertrochanter BMD	0.22 (0.13, 0.39) <0.0001	0.23 (0.12, 0.44) <0.0001	0.18 (0.08, 0.38) <0.0001
Cancer mortality			
Total femur BMD	0.46 (0.24, 0.88) 0.0186	0.22 (0.10, 0.48) 0.0001	0.19 (0.08, 0.49) 0.0005
Femur neck BMD	0.38 (0.18, 0.82) 0.0133	0.28 (0.11, 0.67) 0.0042	0.27 (0.10, 0.74) 0.0109
Trochanter BMD	0.58 (0.27, 1.22) 0.1487	0.25 (0.10, 0.60) 0.0019	0.29 (0.11, 0.77) 0.0136
Intertrochanter BMD	0.45 (0.26, 0.77) 0.0038	0.25 (0.13, 0.48) <0.0001	0.21 (0.10, 0.46) <0.0001
Noncancer non-CVD mortality			
Total femur BMD	0.11 (0.07, 0.18) <0.0001	0.13 (0.07, 0.23) <0.0001	0.25 (0.13, 0.48) <0.0001
Femur neck BMD	0.08 (0.04, 0.13) <0.0001	0.17 (0.09, 0.33) <0.0001	0.33 (0.16, 0.69) 0.0031
Trochanter BMD	0.09 (0.05, 0.15) <0.0001	0.09 (0.05, 0.17) <0.0001	0.18 (0.09, 0.37) <0.0001
Intertrochanter BMD	0.17 (0.11, 0.25) <0.0001	0.21 (0.13, 0.33) <0.0001	0.35 (0.20, 0.61) 0.0002

Adjust I model adjust for: Age; Gender; Race/ethnicity; Education level; Family income to poverty ratio.

Adjust II model adjust for: Age; Gender; Race/ethnicity; Education level; Family income to poverty ratio; Body mass index; Waist circumference; Serum 25(OH)D concentrations; Hypertension; Diabetes; Smoking status.
