# Supplementary material for: Threshold effects of bone mineral density on mortality risk: a comprehensive analysis of BMI-mediated pathways in older population
Source: Front Endocrinol (Lausanne). 2025 Jul 22;16:1567047. doi: 10.3389/fendo.2025.1567047 (PMC12321544; doi:10.3389/fendo.2025.1567047)
Supplement: Supplementary file 4 [file Table2.pdf]

Supplementary Table 2 Threshold effects of bone mineral density on cause-specific mortality

BMD Site/Outcome:	all-cause mortality	CVD mortality	Cancer mortality	Noncancer non-CVD mortality
Total femur BMD OR (95% CI) P				
Model I[b] One-line slope	0.19 (0.12, 0.30) <0.0001	0.12 (0.05, 0.29) <0.0001	0.19 (0.08, 0.49) 0.0005	0.25 (0.13, 0.48) <0.0001
Model II[c]				
Inflection point (K), °C	0.65	0.65	0.83	0.65
<K	0.00 (0.00, 0.01) <0.0001	0.00 (0.00, 0.05) 0.0009	0.02 (0.00, 0.17) 0.0002	0.00 (0.00, 0.02) <0.0001
>K	0.27 (0.16, 0.43) <0.0001	0.16 (0.06, 0.42) 0.0002	0.47 (0.15, 1.51) 0.2041	0.37 (0.19, 0.75) 0.0054
Slope 2 – Slope 1	187.92 (20.88, 1691.59) <0.0001	226.83 (2.46, 20931.18) 0.0188	19.48 (1.63, 232.12) 0.0188	236.13 (13.24, 4210.02) 0.0002
LRT[d]	<0.001	0.033	0.022	<0.001
Femur neck BMD OR (95% CI) P				
Model I[b] One-line slope	0.26 (0.15, 0.43) <0.0001	0.15 (0.06, 0.43) 0.0003	0.27 (0.10, 0.74) 0.0109	0.33 (0.16, 0.69) 0.0031
Model II[c]				
Inflection point (K), °C	0.66	0.75	0.64	0.67
<K	0.02 (0.00, 0.06) <0.0001	0.06 (0.01, 0.33) 0.0011	0.01 (0.00, 0.10) 0.0005	0.02 (0.00, 0.08) <0.0001
>K	0.67 (0.35, 1.26) 0.2141	0.41 (0.07, 2.26) 0.3067	0.63 (0.19, 2.03) 0.4384	1.17 (0.46, 2.97) 0.7400
Slope 2 – Slope 1	40.31 (8.66, 187.70) <0.0001	6.64 (0.42, 103.98) 0.1774	110.02 (3.63, 3331.53) 0.0069	67.54 (8.61, 530.03) <0.0001
LRT[d]	<0.001	0.183	0.010	<0.001
Trochanter BMD OR (95% CI) P				
Model I[b] One-line slope	0.17 (0.11, 0.29) <0.0001	0.11 (0.04, 0.29) <0.0001	0.29 (0.11, 0.77) 0.0136	0.18 (0.09, 0.37) <0.0001
Model II[c]				
Inflection point (K), °C	0.68	0.5	0.62	0.72
<K	0.04 (0.02, 0.11) <0.0001	0.00 (0.00, 0.05) 0.0009	0.03 (0.00, 0.31) 0.0036	0.05 (0.02, 0.16) <0.0001
>K	0.54 (0.25, 1.15) 0.1115	0.17 (0.06, 0.49) 0.0010	0.63 (0.18, 2.14) 0.4587	0.94 (0.26, 3.34) 0.9218
Slope 2 – Slope 1	12.21 (3.27, 45.64) 0.0002	340.17 (2.73, 42457.92) 0.0179	22.83 (1.14, 455.40) 0.0405	17.37 (2.61, 115.60) 0.0032
LRT[d]	<0.001	0.026	0.045	0.004

Intertrochanter BMD OR (95% CI) P				
Model I[b] One-line slope	0.26 (0.17, 0.38) <0.0001	0.18 (0.08, 0.38) <0.0001	0.21 (0.10, 0.46) <0.0001	0.35 (0.20, 0.61) 0.0002
Model II[c]				
Inflection point (K), °C	0.77	0.77	1.01	0.77
<K	0.01 (0.00, 0.04) <0.0001	0.01 (0.00, 0.33) 0.0102	0.05 (0.01, 0.23) <0.0001	0.01 (0.00, 0.05) <0.0001
>K	0.34 (0.23, 0.52) <0.0001	0.22 (0.10, 0.49) 0.0002	0.47 (0.16, 1.35) 0.1620	0.52 (0.29, 0.93) 0.0280
Slope 2 – Slope 1	49.01 (8.58, 279.86) <0.0001	23.52 (0.53, 1048.24) 0.1031	8.73 (1.20, 63.74) 0.0326	89.84 (9.41, 857.67) <0.0001
LRT[d]	<0.001	0.129	0.036	<0.001

[a]Adjusted variables: Age; Gender; Race/ethnicity; Education level; Family income to poverty ratio; Body mass index; Waist circumference; Serum 25(OH)D concentrations; Hypertension; Diabetes; Smoking status.

[b] Linear analysis, P-value <0.05 indicates a linear relationship.

[c] Non-linear analysis.

[d] P-value <0.05 means Model II is significantly different from Model I, which indicates a non-linear relationship.

Abbreviations: OR, odds ratio; CI, confidence interval; LRT, logarithmic likelihood ratio test.
